# Biodegradable Active Packaging as an Alternative to Conventional Packaging: A Case Study with Chicken Fillets

**DOI:** 10.3390/foods10051126

**Published:** 2021-05-19

**Authors:** Jawad Sarfraz, Anlaug Ådland Hansen, John-Erik Haugen, Trung-Anh Le, Jorunn Nilsen, Josefine Skaret, Tan Phat Huynh, Marit Kvalvåg Pettersen

**Affiliations:** 1 Nofima-Norwegian Institute of Food, Fisheries and Aquaculture Research, NO-1431 Ås, Norway; anlaug.hansen@nofima.no (A.Å.H.); john-erik.haugen@nofima.no (J.-E.H.); josefine.skaret@nofima.no (J.S.); 2 Laboratory of Molecular Science and Engineerng, Åbo Akademi University, 20500 Turku, Finland; trung-anh.le@abo.fi (T.-A.L.); tan.huynh@abo.fi (T.P.H.); 3 Norner AS, Asdalstrand 291, NO-3962 Stathelle, Norway; jorunn.nilsen@norner.no

**Keywords:** biodegradable, active, natural, essential oil, shelf life, antimicrobial, sensory, poultry

## Abstract

Innovative active packaging has the potential to maintain the food quality and preserve the food safety for extended period. The aim of this study was to discover the effect of active films based on commercially available polylactic acid blend (PLA_b_) and natural active components on the shelf life and organoleptic properties of chicken fillets and to find out; to what extent they can be used as replacement to the traditional packaging materials. In this study, commercially available PLA_b_ was compounded with citral and cinnamon oil. Active films with 300 µm thickness were then produced on a blown film extruder. The PLA_b_-based films were thermoformed into trays. Fresh chicken breast fillets were packed under two different gas compositions, modified atmosphere packaging of 60% CO_2_/40% N_2_, and 75% O_2_/25% CO_2_ and stored at 4 °C. The effect of active packaging materials and gas compositions on the drip loss, dry matter content, organoleptic properties, and microbial quality of the chicken fillets were studied over a storage time of 24 days. The presence of active components in the compounded films was confirmed with FTIR, in addition the release of active components in the headspace of the packaging was established with GC/MS. Additionally, gas barrier properties of the packages were studied. No negative impact on the drip loss and dry matter content was observed. The results show that PLA_b_-based active packaging can maintain the quality of the chicken fillets and have the potential to replace the traditional packaging materials, such as APET/PE trays.

## 1. Introduction

Active packaging based on natural active components has gained much attention during last decade. A packaging is termed “active” when it provides functions beyond the traditional protection and inert barrier to the outside environment [1]. The active packaging consistently interacts with the food and the surrounding environment of the food inside package. In recent years, a lot of research efforts have been put in developing active packaging solutions. These packaging with active components, such as antimicrobials, antioxidants, O_2_ scavengers, CO_2_ emitters/absorbents, moisture regulators, flavor releasers, and absorbers can delay or stop microbial, enzymatic, and oxidative spoilage [2,3,4]. Based on the source, the active (antioxidant, antimicrobial) components can be classified in to natural or synthetic. Natural plant-based materials, including essential oils (EO) and their major components, have known antimicrobial and antioxidant properties. EO have been in use for long time as flavoring agents for food [5]. Compounds such as linalool, cinnamaldehyde, carvacrol, thymol, and citral, which are major components of cilantro, cinnamon, oregano, thyme, and lemongrass EO, respectively, have the potential to replace some of the synthetic additives. The antimicrobial activity of the EO can be affected by the pH, fat, and protein content of the product [6]. It has been reported that the EO are more effective against Gram positive bacteria compared to Gram negative bacteria [7]. The EO disturb the structure and permeability of the cell membrane, they interact with the proteins and enzymes and disturb the important cell functions for example, proton motive force, electron flow, energy regulation, or synthesis of structural components [6,7,8].

Polymeric food packaging materials can be broadly divided into biodegradable and nonbiodegradable. Traditionally nonbiodegradable polymers, such as, polyethylene (PE), polypropylene (PP), polyethylene terephthalate (PET), polyamide (PA), etc., are used in food packaging applications. To reduce the environmental impact from these traditional packaging materials, an alternative approach of developing packaging solutions based on biodegradable materials is being pursued. In recent years, several studies have reported biodegradable and edible coatings with active components such as mint, grape fruit peel extracts, thymol, ferulic acid, caffeic acid, and tyrosol incorporated in—for example—guar gum, gelatin, chitosan, polyvinyl alcohol, or polylactic acid (PLA) matrix with profound antioxidant properties [9,10,11]. Similarly, the antibacterial properties of various edible coatings and biodegradable packaging with active components based on essential oils for example lemongrass, cinnamon, ziziphora clinopodioides, and/or nanoparticles (e.g., copper oxide, zinc oxide, etc.) incorporated in—for example—alginate, Poly (3-hydroxybutyrate-co-3-hydroxyvalerate), chitosan, sodium alginate-carboxymethyl cellulose, or PLA matrix have been reported [12,13,14,15,16].

PLA is a promising biodegradable and renewable thermoplastic polyester. However, it is hygroscopic in nature, brittle, and less heat tolerant, which makes it less attractive for demanding applications such as food packaging [17]. These properties are greatly improved in the new commercially available PLA blend (PLA_b_) materials by the addition of copolyester. Though, PLA films incorporating EO and their major components have been reported in the literature with promising antimicrobial results. These active PLA films were very seldom applied to the real products and when applied, the antimicrobial properties are studied for a short period of time [18,19,20]. This knowledge gap related to the effect of PLA-based active materials on the real products specially during their entire shelf life, limits the use of these materials in practical applications.

The main objective of this study was to explore the effect of active films based on commercially available PLA_b_ and natural active components on the shelf life and organoleptic properties of chicken fillets and to find out to what extent they can be used as replacement to the traditional packaging materials. The active films have been developed by incorporating citral and cinnamon oil in different amounts in the polymer matrix.

## 2. Materials and Methods

### 2.1. Compounding and Film Blowing

The two additives, cinnamon oil and citral were mixed into Ecovio F2224 using an extruder type W&P26, ZSK MCC with L/D 40 (where L is length and D is diameter) from Coperion GmbH, Germany. No other components were added to the blend. The extrusion temperature was set to 180 °C with a flat profile. The temperature profile on the extruder was increasing from 163 °C in the feeding zone to 183 °C at the die. The extrusion speed was 60 rpm, the output 30 kg/h and N_2_ flushing was used. The pure Ecovio F2224 was run through the compounder using the same condition. Compounds of Ecovio F2224 with 4% cinnamon oil as well as 4% citral were made. In addition, a sample with both 2% cinnamon oil and 2% citral was made. The EO (cinnamon and citral) concentrations were selected based on previously reported levels in the literature [21,22] and to limitations in processing parameters as reported by others [22].

From the compounds, 300 µm films were produced on a blown film extruder model E 25, BL180/400 from Collin Lab & Pilot Solutions GmbH, Germany. The temperature profile on the extruder was set from 160 °C to 183 °C in the different zones, and the extrusion speed was 100 rpm. The die gap was 1.5 mm and the blow-up ratio was 1:3.

### 2.2. FTIR

The Attenuated Total Reflectance Fourier Transform infrared (ATR–FTIR) spectra were recorded using a Thermo Scientific Nicolet iS50 Spectrometer. The instrument is equipped with a diamond crystal and a pressure gauge. The spectra were measured from 700 to 2000 cm^−1^ with a resolution of 4.0 cm^−1^ and averaged from 64 scans.

### 2.3. Packaging and Storage Conditions

Fresh chicken breast fillets were obtained from a Norwegian producer and the packaging was performed within 48 h after slaughtering by 10 different packaging combinations (PC) as listed in Table 1. Selection of fillets to the packages was randomized to achieve an equal/representative number of total viable count of bacteria on the surface. The chicken fillets were packed using a Multivac R145 thermoformer (Multivac, Wolfertschwenden, Germany). The films were thermoformed to a depth of 3.5 cm at 100 °C for 2 s, after warming for 2.5 s. Bottom webs were sealed to a biaxially oriented polyester top web with an amorphous polyester heat seal (Mylar OL 40, Petroplast, Neuss, Germany). The sealing was done at 150 °C for 2 s. The packages were evacuated to 10 mbar before filling with gas for the modified atmosphere packaging (MAP). MAP was done with two different gas mixtures, 60% CO_2_/40% N_2_ and 75% O_2_/25% CO_2_ (AGA, Oslo, Norway). Pre-formed APET/PE trays (K 2187-1U, Faerch plast, Holsebro, Denmark) were used as a positive control. A Multivac T200 tray sealer (Multivac, Wolfertschwenden, Germany) was used for packaging of the APET/PE trays. The APET/PE trays were sealed with the same top web as mentioned above. Two fillets weighing approximately 320 g were packed in the PLA-based trays with volume of 980 mL, and two fillets weighing approximately 250 g were packed in APET/PE trays with volume of 825 mL, resulting in initial gas volume to product volume ratio (g/p ratio) in the range of 2.2 to 2.5. The chicken breast fillets were stored in dark at 4 °C with samples taken after 0, 8, 14, 20, and 24 days of storage.

### 2.4. Head Space Gas Chromatography/Mass Spectrometry (GC/MS)

Gas samples were taken by using a syringe to draw 120 mL of the headspace in the packages through an adsorbent tube packed with Tenax GR for trapping the volatile compounds. The adsorbent tubes were desorbed at 280 °C for 7 min in a Markes Thermal Desorber and transferred to an Agilent 6890 GC with an Agilent 5973 Mass Selective Detector (EI, 70 eV). The volatiles were separated on a DB-WAXetr column (30 m, 0.25 mm i.d., 0.5 µm film) with a temperature program starting at 30 °C for 10 min, increasing 1/min to 40 °C, 3/min to 70 °C, and 6.5/min to 230 °C, hold time 5 min. The peaks were integrated, and compounds tentatively identified with HP Chemstation software and NIST 2011 Mass Spectral Library. The volatile compounds are expressed in arbitrary units of the deconvoluted component of the peak area. The result shown are an average of three replicates.

### 2.5. Gas Transmission Rates

The oxygen transmission rate (OTR) for the different packages was found by the ambient oxygen ingress rate method (AOIR) [23] and the carbon dioxide transmission rate (CO_2_TR) was determined as described by Larsen and Liland (2013) [24]. The gas transmission rates were reported as ml gas/package/day. The OTR and CO_2_TR were measured at 4 °C, approximately 40% RH. The result shown are an average of four replicates.

### 2.6. Drip Loss

Drip loss was determined by initially weighing empty packages (A), packages with the fillets at the time of packaging (B), and at each sampling time weighing of (C) the packages including the drip loss liquid after removing the chicken. The amount of drip loss was found by calculating the increase in weight of the packages (C-A) and divided by the initial weight of the chicken: (C-A)/(B-A). Results are given as the percentage (%) of initial muscle weight and refer to the corresponding liquid loss from the meat sample. The result shown are an average of three replicates.

### 2.7. Dry Matter

Dry matter in chicken breast fillets was determined at each sampling time. The water content was determined in difference of weight before and after oven-drying at 105 °C for 16–18 h. The chicken fillets were homogenized and approximately 6 ± 0.5 g was weighed and stored in petri dishes (diameter 90 mm) before drying. After drying, the petri dishes were cooled to room temperature in an exicator before weighing. The dry matter was calculated as 100% minus water content. The result is given as average of three replicates.

### 2.8. Microbiology

Selected microbiological analyses were performed at the time of packaging (five replicates) and at each sampling time; after 8, 14, 20, and 24 days of storage (three replicates) as explained earlier [25]. Briefly, from the surface of the fillet, samples of 3 × 3 cm^2^ and 1 cm depth were cut with a sterile scalpel, weighed, and added approximately 90 mL peptone water to a 1/10 dilution and run in a stomacher for 60 s.

Appropriate 10-folds dilutions were spread by using Whitley automatic spiral plater (WASP) (Don Whitley Scientific Ltd. Bingley, England) in duplicate on:

1. Plate count agar (PCA; Difco, Difco Laboratories, Detroit, MI, USA) for total viable counts (TVC): incubation temperature 30 °C, 72 h, aerobic incubation. (NMKL No. 86);
2. De Man, Sharpe, and Rogosa agar (MRS; Oxoid, Unipath Ltd., Basingstoke, Hampshire, UK,) for lactic acid bacteria (LAB): 25–30 °C, 48 h, aerobic incubation. (NMKL No. 140);
3. Streptomycin thallous acetate actidione (STAA) agar base (CM 0881 with selective supplement SR 0151E, Oxoid, Hampshire, England) for *Brochothrix thermosphacta*; 25 °C for 48 h, aerobic incubation. (NMKL No. 141);
4. Violet Red Bile Glucose Agar (VRBGA, Oxoid, Hampshire, UK) for *Enterobacteriace*; 37 °C, 24–48 h, semi-aerobic conditions, cells embedded in agar with sterile overlay. (NMKL No. 144).

Microbial counts are expressed as colony forming units (cfu) per g.

### 2.9. Sensory Analysis

To describe the objective perception of the various samples, a trained panel consisting of 10 assessors, performed a Quality Descriptive Analysis, ISO 13299:2016 (E) of the samples after 14 days of storage. The panelists have been trained according to recommendations in ISO 8586:2012 (E). The sensory laboratory has been designed according to guidelines in ISO 8589:2007 (E) with separate booths and electronic registration of data (Eye Question, v. 3.8.6, Logic 8, Elst, The Netherlands), standardized light, and a separate ventilation system.

The samples were taken from the cold storage room for tempering two hours prior to evaluation and cut up, alongside, and put into bowls 30 min before evaluation. The samples were 18 ± 2 °C when serving. Raw chicken, one half of a fillet was first served to the assessors in transparent plastic cups covered with lid for evaluation of the odor. Then, the samples were taken out of the cups and placed on a white cardboard plate, and the appearance of the samples was evaluated. Finally, the assessors evaluated texture by pressing the sample on the thickest part with two fingers (using plastic gloves). The coded samples were served in blind trials randomized according to sample, assessor, and replicate.

The panelists evaluated the samples in duplicate, during five sessions with at least fifteen minutes break between each session. The assessors recorded their results at individual speed on a 15 cm non-structured continuous scale with the left side of the scale corresponding to the lowest intensity and the right side corresponding to the highest intensity. The computer transformed the responses into numbers between 1 = low intensity and 9 = high intensity.

### 2.10. Statistical Analyses

To compare the results obtained from 10 different PC as listed in Table 1, one-way analysis of variance (ANOVA) and comparisons Tukey test (which compares all pairs of groups, while controlling the simultaneous confidence level) was performed at each sampling time. General Linear Model (GLM) was applied with the variables: materials, gas composition, and storage time. Their second order interactions were fitted to the measured responses. Analysis of variance has been conducted in Minitab 18.

The multivariate statistics software package, The Unscrambler^®^ v9.8 (Camom Software AS, Oslo, Norway) was used for the principal component analysis (PCA) and partial least squares regression (PLSR) analysis of the combined chemical and sensory data. Full cross validation (leaving one sample out) and raw and auto scaled preprocessing (variables divide by their standard deviation) of the data were used in the data analysis.

## 3. Results and Discussion

### 3.1. FTIR

**Figure 1:**
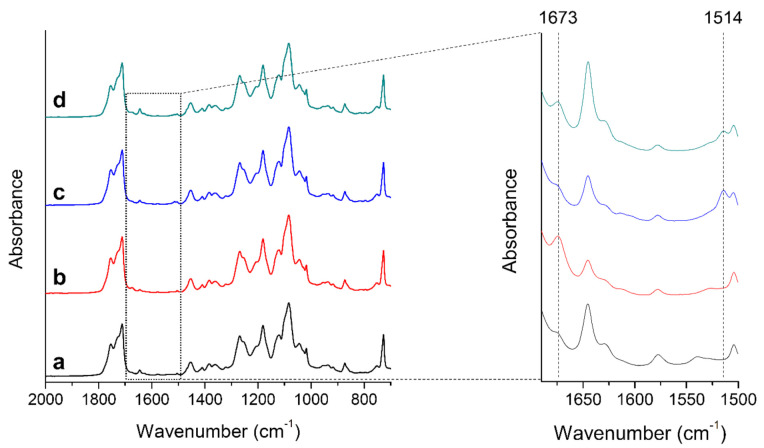
IR spectra of the 300 µm thick PLA_b_ (**a**) before doping and after incorporating (**b**) 4% citral, (**c**) 4% cinnamon oil, and (**d**) 2% citral and 2% cinnamon oil. The right figure is a zoom of all spectra at wavenumber of 1500–1680 cm^−1^ where significant differences can be seen at 1514 and 1673 cm^−1^.

FTIR was performed in order to confirm the presence of active components in the compounded films. Figure 1 show IR spectra of the PLA_b_ film and PLA_b_-based active films. In the PLA_b_-citral sample, the band at 1673 cm^−1^ is particularly noticeable (Figure 1b) due to contribution of the -C=O- stretching vibration of citral [26,27]. On the other hand, the 1514 cm^−1^ band is distinct for the PLA_b_-cinnamon oil (Figure 1c) and is believed to belong to -C=C- stretching vibration of the aromatic ring or N-O stretching (of nitro compound left in cinnamon oil) [28]. The band is quite distinct as it is separated from the band of 1504 cm^−1^ which is for the pure PLA_b_ sample. The IR bands of both citral and cinnamon can also be discriminated for PLA_b_ film containing 2% citral and 2% cinnamon oil (Figure 1d). Interestingly, their intensity is half of 4% citral (for the 1673 cm^−1^ band) and 4% cinnamon (for the 1514 cm^−1^ band).

These results manifest FTIR as a useful tool for both qualitative and semi-quantitative evaluation of the incorporated active components. The success in qualitative measurement is due to the major difference in the chemical formula (with and without the aromatic ring) of citral and cinnamon. Meanwhile, the concentrations of citral and cinnamon incorporated in the PLA_b_ film closely correlate with intensity of the IR bands, and therefore allow to be semi-quantified by FTIR.

### 3.2. Gas Barrier Properties

Typically, for food packaging applications carbon dioxide transmission rate (CO_2_TR) and oxygen transmission rate (OTR) is measured to quantify the gas barrier properties of the polymer.

**Figure 2:**
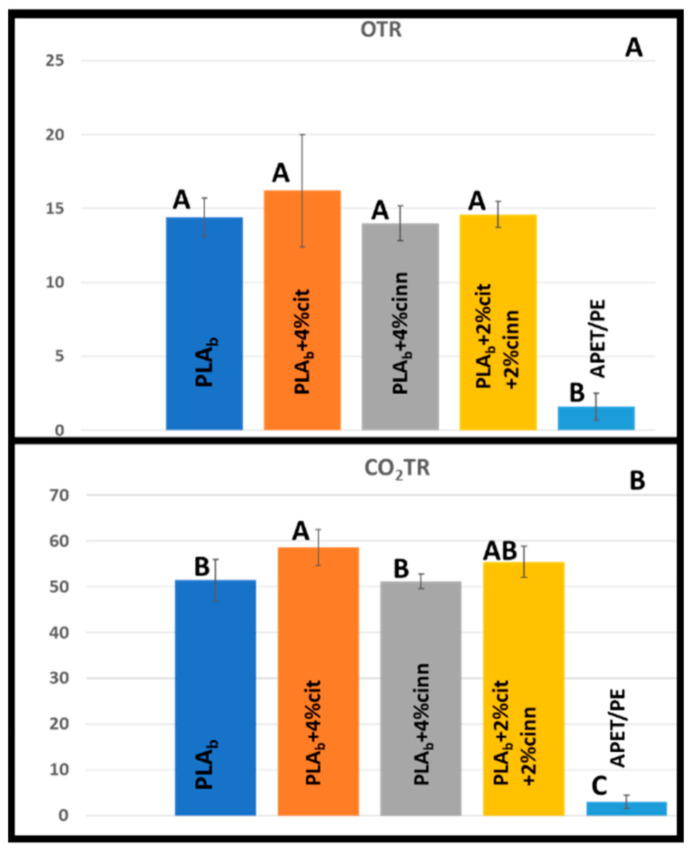
The oxygen transmission rate (**A**) and carbon dioxide transmission rate (**B**) of the PLA_b_, PLA_b_ composites and APET/PE packages. (cit = citral, cinn = cinnamon oil) in ml gas/package/day. Upper case letters represent one-way analysis of variance Tukey comparison, samples which do not share the same letter are significantly different.

The Figure 2A,B shows the OTR and CO_2_TR of PLA_b_-based packages and the reference consisting of APET/PE. The OTR and CO_2_TR of the reference APET/PE package was 1.6 ± 0.9 and 3 ± 1.4 mL/package/day, respectively, while for PLA_b_ package the OTR was 14.4 ± 1.3 and CO_2_TR was 51.4 ± 4.6 mL/package/day. Zeid et al. reported OTR for 112 µm PLA to be 865.9 ± 38.9 mL/m^2^/day atm [29]. However, OTR is dependent on several aspects such as thermoforming and material thickness, in addition to additives.

Figure 2A,B shows that the PLA_b_ based packages has relatively poor barrier properties compared to the APET/PE as expected. According to one-way ANOVA, there was no significant difference in the OTR of the PLA_b_ based packages, which implies that the oxygen barrier properties of PLA_b_ were not significantly affected by the incorporation of the active components (citral and/or cinnamon oil) corresponding to other findings [28]. However, APET/PE package has, as expected, lower OTR compared to the PLA_b_ based packages (Figure 2A). On the other hand, the addition of citral in the PLA_b_ matrix has slightly increased the CO_2_TR. (Figure 2B). In contrast, the addition of cinnamon oil did not significantly change the CO_2_TR. The differences in OTR for the PLA_b_ and APET/PE packages in our study were approximately 10-fold. In the literature 100× higher oxygen permeability for PLA has been reported compared to PET. [30] For PLA, 216 cm^3^.mm/m^2^.day.atm and for PET, 1.2–2.4 cm^3^.mm/m^2^.day.atm were reported at 23 °C [21]. Similar OTR trends were also reported for PLA and APET/PE [31,32]. However, as described by Pettersen et al., OTR measured for PLA based pouch with 40 µm thickness was 4.75 mL O_2_/package/day as compared to 0.031 mL O_2_/package/day for thermoformed trays made of 550 µm APET/PE measured at 6 °C [31].

Typically, APET/PE material is used for MAP of chicken breast fillets and when packed under 60% CO_2_/40% N_2_ (commonly applied gas composition in Norway), the shelf life of approximately 3 weeks can be achieved for chicken fillets [33].

The oxygen content in the PLA_b_ based packages with chicken stored in 60% CO_2_/40% N_2_ increased to almost 2% after 14 days of storage, while the level was only 0.1% in the APET/PE reference package during the same time. As expected, the CO_2_ concentration in all packages decreased during storage due to solubility of CO_2_ into the meat. In PLA_b_ based packages the level of CO_2_ decreased from 60% to approximately 30% after 7 days of storage, with further reduction to around 20% at the end of storage time and for APET/PE the level of CO_2_ decreases to 45% after 7 days of storage and stayed almost at the same level to the end of storage time.

In packages with chicken store in 75% O_2_/25% CO_2_ atmosphere the oxygen content slightly increased in all packages (up to 80% after 14 days of storage in the PLA_b_-based packages), followed by a reduction to 70–75%. The level of CO_2_ was reduced from initial 22% to approximately 14% after 14 days of storage and an increase to 17–19% after 24 days of storage. For APET/PE the content CO_2_ decreased somewhat (from 22 to 20%) after 7 days and increased to 25% at the end of storage time.

The presence of oxygen in the package can have many adverse effects on the product including oxidative spoilage, off odor, and discoloration which limits the shelf life of the product. On the other hand, the use of CO_2_ in MAP can suppress the growth of common spoilage bacteria [34], thus improving the shelf life and food safety of the product.

### 3.3. Drip Loss and Dry Matter

Chicken fillets are prone to drip loss and several factors, such as time of deboning, refrigerator time, and packaging environment, may affect the liquid loss [35]. In order to maintain an attractive product appearance as well as to avoid reduction of sensory quality, such as juiciness, drip loss from the product should be minimized. Earlier findings have shown that MAP with relatively high CO_2_ concentration (≥40%) increases the drip loss. This increase in drip loss has been explained by the reduction in pH, resulting in the decrease in water holding capacity of proteins [36,37]. However, Pettersen et al., reported no significant differences in drip loss for chicken stored in high CO_2_ concentration (60% CO_2_/40% N_2_) compared to lower concentration (25% CO_2_/75% O_2_) [38]. Holck et al., have also shown that the drip loss is not only related to the content of CO_2_ in the head space, as high content of CO_2_ with addition of a CO_2_ emitter resulted in lower drip loss compared to samples with lower content of CO_2_ [25,38]. The absorption of CO_2_ at the surface of the product may result in under pressure in the package, which may increase the drip loss. However, it also depends on the packaging design and degree of filling (gas/product ratio). Furthermore, it has been reported in the literature that increased drip loss will take place in rigid packages compared to flexible ones [39].

**Figure 3:**
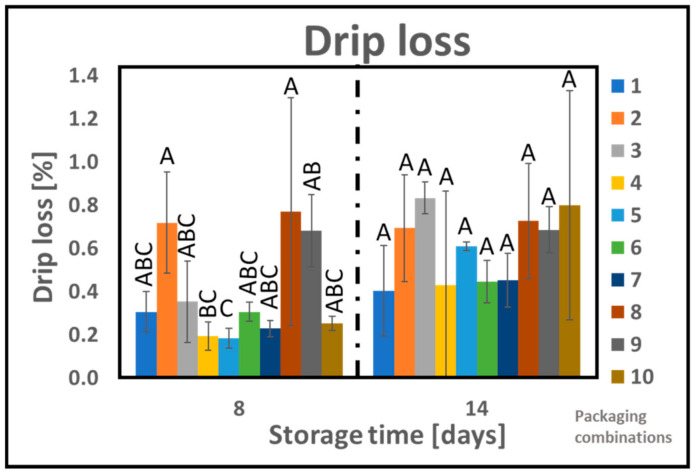
Drip loss of chicken samples as a function of storage time for different packaging combinations. One-way analysis of variance Tukey comparison is performed separately at different sampling points. Tukey group is represented by upper case letters, samples which do not share the same letter are significantly different.

Chicken fillets were stored by applying ten different packaging combinations (1–10), as listed in Table 1. The drip loss from the product has been measured and the results are shown in Figure 3. According to GLM (with variables: material, gas composition, and storage), neither the material nor the gas composition has any significant effect on the drip loss in our study (Table S1). On the other hand, storage time as well as the interaction between material and gas composition has a significant effect on the drip loss as explained by 40.5% and 7.1% of the variance, respectively. Figure 3 shows that the drip loss increased slightly with the storage time. Nevertheless, a maximum drip loss of around 2% was measured at the end of 24 days. These results are in agreement with the previously reported values in the literature under similar conditions. Holck et al. has reported a drip loss of around 2.5% for chicken fillets stored under MAP (60% CO_2_/40% N_2_) at the end of 26 days. According to one-way Anova performed for each sampling time, only after 8 days of storage, significant differences between some of the packaging combinations of PLA_b_ were observed, as shown in Figure 3. Furthermore, storage of chicken in APET/PE with 60% CO_2_ (PC 9) resulted in higher drip loss compared to PC 5 (PLA_b_ + 4% cinnamon oil with 60% CO_2_) and were placed in different Tukey groups as shown in Figure 3. For all other sampling time (14, 20, and 24 days) no significant differences between the samples were observed. Figure 3 shows that the PLA_b_-based active packaging did not contribute to any increase in the drip loss which is further confirmed by the GLM analysis. Pure PLA is hygroscopic in nature [17], which could be of importance when studying the interaction of this material with high water content products. In this regard, drip loss, appearance, and surface dryness are important parameters to study [40].

The moisture and dry matter content are important guides for the meat quality. The dry matter content is positively correlated with total nutrient content [41]. Shaarani et al. (2006) reported moisture content in broiler meat being as high as 76% [42]. The dry matter content results are shown in Figure S1. According to statistical analysis, GLM, material, and storage time has a significant effect on the dry matter content and explained variance of 8.8% and 12.3%, respectively. However, residual variation was relatively high, 65% and residual R^2^ (adj) only 15.5%, meaning that much of the variance was not explained by the model. It can be seen (Figure S1) that at any given sampling time, the variation in dry matter content between the samples is less than 1%, this difference is probably not of practical importance. According to one-way Anova performed for each sampling time (8, 14, 20, and 24 days), no significant differences between the samples were observed. Furthermore, the dry matter content of 24.5 ± 1% independent of packaging combination is in agreement with the reported values in the literature [42].

### 3.4. Microbiological Growth

The initial level of bacteria in the fresh chicken breast fillets measured as TVC was about 2.8 log cfu/g at the time of packaging.

**Figure 4:**
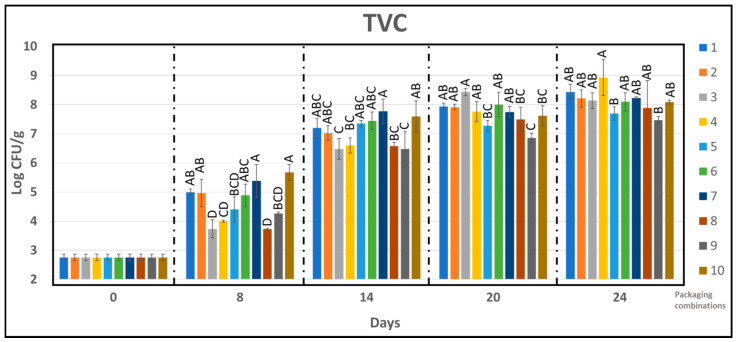
The effect of packaging combinations on the total viable counts (log cfu/g) in chicken samples during storage time of 24 days. One-way analysis of variance Tukey comparison is performed separately at sampling point 8, 14, 20, and 24. Tukey group is represented by upper case letters, samples which do not share the same letter are significantly different.

Figure 4 shows the effect of the ten different packaging combinations on the microbial quality measured as TVC of the chicken fillets. According to the GLM (Table S1), storage time has significant effect on the microbial growth, as expected. TVC in all samples increased from the initial level of about 2.8 log cfu/g to above 6 log cfu/g during the first 14 days of storage (Figure 4). According to the literature, spoilage can be found at total bacterial count levels of above 7 log cfu/g, which indicates that samples stored in some of the packaging combinations presented had passed its microbiological shelf life already after 14 days of storage [37,43,44]. During the first 14 days of storage, under 60% CO_2_/40% N_2_ all the chicken samples packed in PLA_b_ and PLA_b_ composites have higher growth of bacteria compared to the reference APET/PE. However, opposite trend was observed under 75% O_2_/25% CO_2_.

According to the Tukey pair wise comparison of the TVC at the end of 8 days, lowest level of TVC (3.7 log cfu/g) was obtained for some of the packaging combinations containing essential oils (Figure 4). Furthermore, the TVC results show that the microbiological quality of the chicken meat packed in APET/PE package was no different than those packed in PLA_b_-based active packages despite relatively poor gas barrier properties of these materials.

It can be concluded from the TVC results that, during the first 8 days of storage, some effect of active compounds is obtained as inhibition in the growth of microorganisms has been achieved from PC 3, 4, and 8 (all PLA_b_ with added citral) compared to the positive control (PC 9; APET/PE with 60%CO_2_ / 40%N_2_). It can correspond to the sustained release of active components (further confirmed by GC/MS Section 3.5). PC 3 (PLA_b_ added 4% citral) has significant lower level of TVC compared to neat PLA_b_ (PC 1) and PC 7 (PLA_b_ added 2%Citral +2% cinnamon) after 8 and 14 days of storage. Furthermore, the TVC results from sampling time 14, 20, and 24 days confirmed that PLA_b_-based packaging (PC 3, 4, 5, and 8) are similar compared to the positive control with APET/PE and 60% CO_2_/40% N_2_ (PC 9) in maintaining the microbiological quality of the chicken at different time points. The reduction of antimicrobial activity as a function of storage time for natural antimicrobials has been reported in the literature [45]. As the antimicrobial activity of the essential oils is based on the release of volatile active components. With the passage of time, due to decrease in the concentration of the active components, their antimicrobial activity decreases (further refer to Section 3.5).

**Figure 5:**
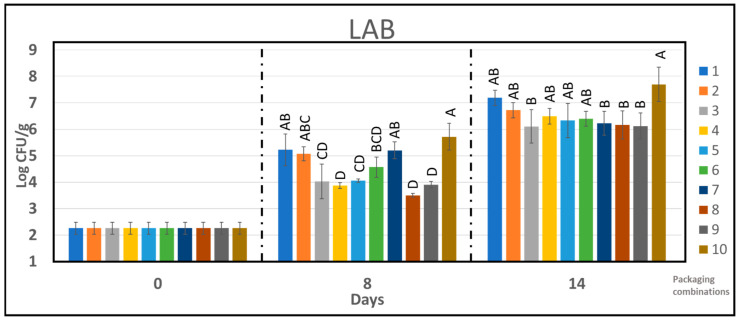
The effect of packaging combinations on the LAB counts in chicken samples during storage time of 14 days. One-way analysis of variance Tukey comparison is performed separately at sampling point 8 and 14 days. Tukey group is represented by upper case letters, samples which do not share the same letter are significantly different.

The growth of three common spoilage bacteria, Lactic acid bacteria (LAB), *Brochothrix thermosphacta,* and *Enterobacteriaceae* were further studied in detail for the chicken samples. Figure 5 shows the effect of different packaging combinations on the growth of LAB. During storage, lactic acid bacteria increased for all samples. This is in accordance with other studies of modified atmosphere packaged poultry products [37,46,47].

Under 60% CO_2_/40% N_2_, the chicken fillets stored in active PLA_b_ composites and APET/PE has similar bacterial load (around 10^6^ cfu/g) after 14 days of storage. However, the LAB count from reference PLA_b_ sample was an order of magnitude higher under the same conditions. Under MAP2 (75% O_2_/25% CO_2_), all PLA_b_ samples have lower LAB counts compared to APET/PE during the 14 days of storage.

After 8 days storage of chicken, PC 4 (PLA_b_ with citral) and PC 8 (PLA_b_ with citral+cinnamon) were similar in suppressing the growth of LAB as compared to control PC 9 and belong to the same Tukey group, as shown in Figure 5. Overall, PLA_b_ containing citral has shown some inhibitory effect towards the growth of lactic acid bacteria after 8 days of storage. However, during further storage the effect diminishes (Figure 5).

**Figure 6:**
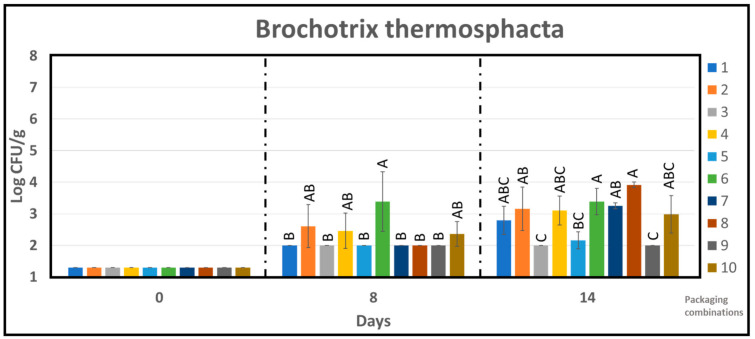
The effect of packaging combinations on the *Brochotrix thermosphacta* counts in chicken samples during storage time of 14 days. One-way analysis of variance Tukey comparison is performed separately at sampling point 8 and 14. Tukey group is represented by upper case letters, samples which do not share the same letter are significantly different.

The count numbers of *Brochothrix thermosphacta* were between 2 to 4 log cfu/g after 14 days of storage, as shown in Figure 6. However, both APET/PE reference and PLA_b_-based packages suppressed the growth of *Brochotrix thermosphacta* under 60% CO_2_/40% N_2_ (Figure 6). Figure 6 further shows that PC 3, 5, and 9 extended the lag phase of *Brochotrix thermosphacta* for up to 14 days under 60% CO_2_/40% N_2_ and during this time the concentration of bacteria remained similar for these materials. It is reported in the literature that increasing concentrations of CO_2_ retards the growth of *Brochothrix thermosphacta* [37].

At 8 days of storage, no significant growth of *Brochothrix thermosphacta* was observed, as shown in Figure 6, and according to one-way Anova, no significant difference between the chicken samples from different PC was obtained except for PC 6 as shown in Figure 6. At 14 days, PLA_b_ in 60% CO_2_/40% N_2_ (PC 3, 5, and 9) had the lowest mean count of *Brochothrix thermosphacta* (same Tukey group). Although significant difference between the samples were observed after 14 days of storage (4 different Tukey group), the mean count was less than 4 log cfu/g for all the samples.

**Figure 7:**
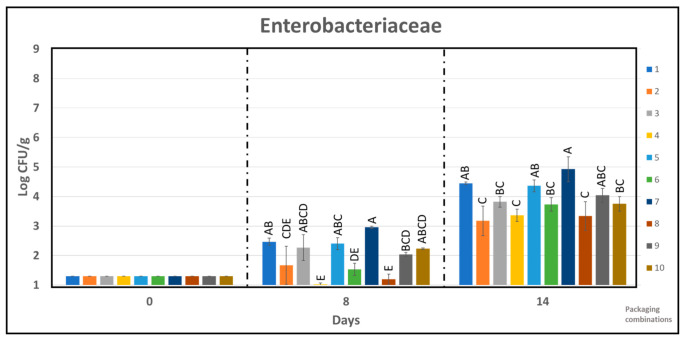
The effect of packaging combinations on the *Enterobacteriaceae* counts in chicken samples during storage time of 14 days. One-way analysis of variance Tukey comparison is performed separately at sampling point 8 and 14. Tukey group is represented by upper case letters, samples which do not share the same letter are significantly different.

Generally, PLAb and PLAb-based active packaging with high O_2_ was more effective in suppressing the growth of Enterobacteriaceae, as shown in Figure 7. During the first 14 days of storage, under 60% CO_2_/40% N_2_ all the chicken samples packed in PLAb and active PLAb composites had higher growth of bacteria compared to the APET/PE (PC 9). However, opposite trend was observed under 75% O_2_/25% CO_2_. The distribution of samples according to different Tukey group at different storage time for Enterobacteriaceae is also shown in Figure 7.

### 3.5. GC/MS

**Figure 8:**
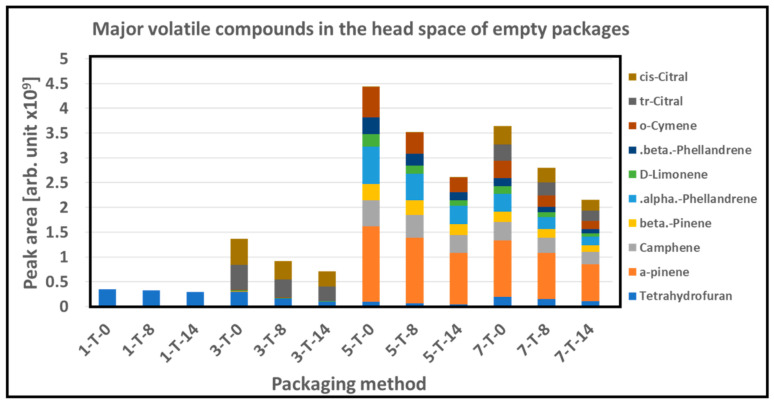
Major volatile compounds in the head space of empty packages with PC 1, 3, 5, and 7 with storage time of 0, 8, and 14 days. (x-T-y; x = packaging method, y = storage time in days).

The GC/MS analysis of the empty packaging shows that volatile compound associated with solvent i.e., tetrahydrofuran was dominated in the PC 1, 3, 5, and 7, as shown in Figure 8. Identified main volatile compounds in the empty packaging and their relative concentrations are compiled in Table S3. PLA_b_ incorporating cinnamon oil (PC 5 and 7) was dominated by the typical major terpenes found in cinnamon oil, i.e., α-pinene, α-phellandrene, o-cymene, and camphene and low levels of the other typical cinnamon oil terpenes [48]. The PLA_b_ containing citral (PC 3 and 7) was dominated by the two major isomers of citral, tr(α)-citral and cis(β)-citral, and low levels of other citral derived terpenes. The levels of these major volatiles in the active packaging materials showed a linear decrease in the range of 20–60% with storage time.

**Figure 9:**
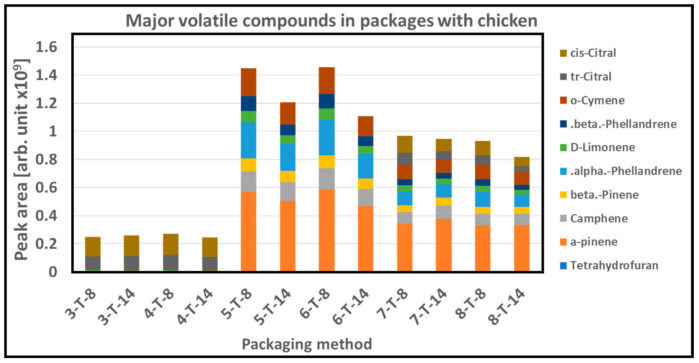
Major volatile compounds in the packaging headspace containing chicken fillets packed with different packaging combinations (PC 3, 4, 5, 6, 7, and 8) after 8 and 14 days of storage. (x-T-y; x = packaging method, y = storage time in days).

Similarly, GC/MS head space analysis of the packaging with chicken samples also shows the strong presence of major terpenes found in cinnamon oil, i.e., α-pinene, β-pinene, α-phellandrene, β-phellandrene, o-cymene, D-limonene, and camphene as well as two major isomers of citral, tr(α)-citral, and cis(β)-citral, as shown in Figure 9. The major cinnamon oil terpenes showed a decrease over 14 days storage in the PLA_b_ + cinnamon oil samples (PC 5 and 6), but in the combined cinnamon oil and citral packaging material (PC 7 and 8), this effect was not that pronounced as shown in Figure 9. The PLA_b_ + citral sample showed a slight increase (PC 3), while the PC 4 showed a slight decrease with storage time in the citral isomers from 8 to 14 days, whereas the combined PLA_b_ cinnamon oil and citral samples (PC 7 and 8), showed a decrease in the citral isomers (Figure 9).

**Figure 10:**
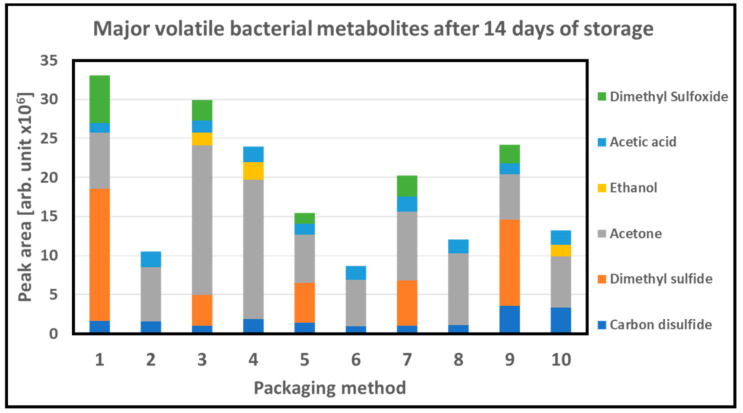
Major volatile bacterial metabolites in the packaging headspace containing chicken fillets packed with different packaging combinations after 14 days of storage.

Figure 10 shows the major volatile bacterial metabolites after the storage time of 14 days. Bacterial secondary metabolites dominated by dimethyl sulphide, acetone, dimethyl sulfoxide, carbon sulphide, acetic acid, and ethanol were detected, as shown in Figure 10. No typical secondary lipid oxidation products from chicken could be found, which could otherwise be expected in the MAP2. These results are in good agreement with the literature. M. Eilamo et al. detected butene, ethanol, acetone, pentane, dimethylsulfide, carbon disulfide, and dimethyl disulfide as primary volatile compounds in gas packed poultry meat with gas composition of oxygen 0–4%, carbon dioxide 20, 50, and 80% with nitrogen being the balancing gas [49].

Upon microbial metabolism, generally volatile organic compounds are produced via two biochemical pathways: glycolytic and proteolytic. Ethanol can be produced from carbohydrates via anaerobic fermentation, or via deamination and decarboxylation of amino acids. In meat, the ethanol production is often attributed to the metabolic activity of lactic acid bacteria. [50,51,52] Sulfides are produced through proteolytic pathway and their production is ascribed to the metabolic activity of *Pseudomonas*, *Enterobacter*, Acinetobacter/Moraxella, and Alteromonas putrefaciens [53].

Furthermore, our results indicate a difference in spoilage between the packaging gases with high levels of dimethyl sulphide and dimethyl sulfoxide for the MAP1 variants compared to the MAP2 variants. This also corroborates with the literature. Dimethyl sulfide, dimethyl disulfide, dimethyl trisulfide, and carbon disulfide has been reported as volatile biomarkers in chicken hindquarters packed under a modified atmosphere [49,54] however under aerobic conditions they are not suitable for early spoilage detection [51]. It can be further seen in Figure 10 that sample from active-PLA_b_ based packaging has significantly lower levels of dimethyl sulfide. The higher levels of acetone found in the citral containing PLA_b_, may partly be ascribed to the oxidation of citral, since acetone is an oxidation product of citral.

Detected major volatile compounds in the packaging with chicken samples with their relative concentrations are listed in Table S4.

### 3.6. Sensory Profile

**Figure 11:**
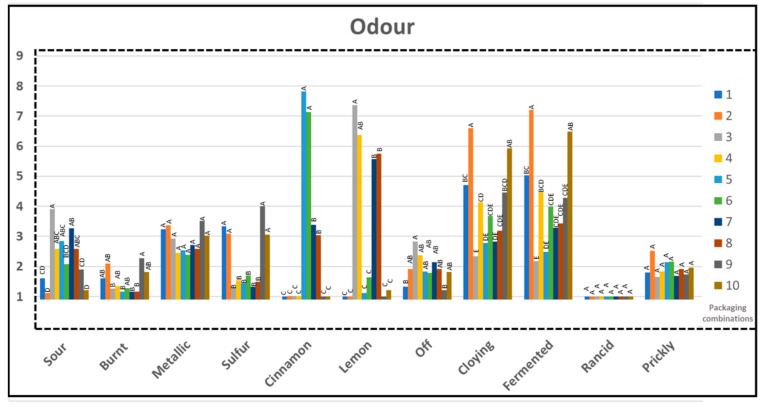
Mean intensities of the assessed odor attributes of all samples after 14 days of storage. Tukey group is represented by upper case letters, samples which do not share the same letter are significantly different.

The chicken samples stored for 14 days were analyzed for odor, appearance, and texture by a trained sensory panel with ten professional assessors using a quantitative descriptive method, ISO 13299:2016E. Definition of sensory attributes is provided in Table 2. ANOVA of the sensory evaluation data revealed that there were significant differences between the chicken variants for all the odor attributes, except for metallic, rancid, and prickly as shown in Figure 11. Odor intensity below 3 is considered low and is generally believed to be not recognizable by majority of the consumers. For appearance and texture attributes, there were statistically significant differences for color hue, color intensity, and gloss. (Table S2) In general, there were greater variations between the samples for the odor attributes than for the appearance and the texture attributes. In addition, surface degradation or surface slime formation was not seen on any of the samples stored with different PC when analyzed at the end of 14 days. Along with oxidative spoilage, the generation and accumulation of metabolic by-products upon enzymatic degradation of food can also lead to discoloration, texture change and development of off-odor. Offensive odor is developed in meat when bacterial flora at the surface exceeds 10^7^ cfu/cm^2^ and above10^8^ cfu/cm^2^ the surface becomes slimy [37,43,44,55]. Interestingly no differences were recorded for dried appearance, dried texture, and firmness. PLA is hygroscopic and is expected to have a drying effect on the product with high water content, such as poultry [17,32]. However, ecovio^®^ F2224 used in this study is a compound of biodegradable copolyester ecoflex^®^ F Blend and polylactic acid (PLA, NatureWorks^®^), which explains the improved properties of the material.

Principal component analysis (PCA) showed that PCA1 and PCA2 described 47 and 23% of the variation among the samples, respectively (Figure S2 ). Lemon and sour odor versus fermented, cloying, and sulfur odor described most of the variations along PCA1 and PCA2. The Biplot further shows similarities in the negative odor attributes between the samples stored in PC 1, 2, 9, and 10 (reference PLA_b_ and APET/PE) with good correlation to cloying, fermented, and sulfur odor (Figure S2). On the opposite direction the PLA_b_ variants with additives correlated to sour odor (PC 3, 4, 7, 8), lemon (PC 3, 4, 7, 8), and cinnamon odor (PC 5, 6).

Sour odor is usually experienced positively by the consumer and is related to a fresh chicken with good quality. On the contrary, fermented, cloying, burnt, sulfur, metallic, off-odor, rancid, and prickly odors are experienced as negative attributes and are related to spoiled chicken with a poor quality. Negative odor attributes cloying and fermented received high scores for samples packed in PC 1, 2, 4, 6, 9, and 10. The mean value for sulfur odor was more than 3 for chicken samples from PC 1 and 9 which can be related to the amount of detected dimethyl sulfide (Figure 10). It can be noted that although dimethyl sulfide was detected for samples from PC 3, 5, and 7 (Figure 10), mean sensory score for sulfur was however much lower for these samples compared to samples stored in PC 2 and 10. It can be implied that the additive (citral and cinnamon oil) might have partially masked the negative sulfur odor in this case.

### 3.7. Volatiles/Sensory

The results from a PLS regression model based on the volatile compounds (x-data) and sensory data (y-data) showed the following relationship between the volatile compounds and the respective sensory odor attributes: As would be expected, the odor attribute sulfur is associated with the volatile sulfur compounds (dimethyl sulfide, carbon disulfide and dimethyl sulfoxide) and to a lesser extent, also the attributes burnt and metallic are associated with these substances, which may be due to the fact that these two attributes may have burnt and metallic notes. Cinnamon odor is strongly associated with tr-cinnamaldehyde, in addition to several other of the sweet, spicy, and woody note terpenes (eugenol, α-thujene, camphene, d-3-carene, caryophyllene, α-copaene, α-humulene, β-linalool, α- and β-pinene, safrole). Also, the solvents toluene and styrene from the packaging material was associated with cinnamon odor, but they should not contribute significantly to the cinnamon odor due to their higher odor thresholds compared to the terpenes [56,57]. The sour and lemon odors are associated with typical citrus and sour note compounds such as the major citral tr and cis isomers, p-cymene and to some extent acetone and acetic acid. Also, off-odor is associated with these substances, which is difficult to explain. However, D-limonene, which has a citrus like character, is associated with cinnamon. But it must be emphasized, that the odor character of the terpene may vary with concentration, and besides, also masking of some odor compounds may also occur due to interactions and synergistic and antagonistic effects between odor compounds. And their respective odor threshold would need to be taken into consideration to be able to explain the relationship between the sensory odor of the chemical substances. The volatile compounds showed, however, a significant positive correlation (PLSR) with several of the odor attributes: lemon odor (r = 0.97, *p* < 0.001, *n* = 14), cinnamon odor (r = 0.90, *p* < 0.001, *n* = 14), sulfur odor (r = 0.86, *p* < 0.001, *n* = 10), off (r = 0.88, *p* < 0.001, *n* = 10), and burnt (r = 0.74. *p* < 0.001, *n* = 14).

## 4. Conclusions

The chicken fillets packaged with active PLA_b_ materials were preserved as well as fillets packaged with the APET/PE materials, despite 10-fold higher oxygen transmission rate of the PLA_b_ compared to the APET/PE packages. No negative impact on the drip loss and dry matter content was observed from the active PLA_b_ composites. The antimicrobial effect of the essential oils was pronounced during the first 8 days of storage. Active PLA_b_-based materials especially those with citral inhibited the growth of microorganisms compared to the positive control (PC 9), which is related to the sustained release of active components as confirmed by GC/MS. Furthermore, the TVC results from sampling time 14, 20, and 24 day confirmed that PLA_b_-based packaging with PC 3, 5, and 8 are similar in maintaining the microbiological quality of the chicken as compared to the positive control (PC 9). However, relatively high level of bacteria (TVC) was measured already after 14 days of storage in all samples. The release of active volatile compounds (terpenes) from the packaging materials has been confirmed by GC/MS. Sulfur compounds derived from spoilage bacteria flora were also detected and it can be indicated that the additives (citral and cinnamon oil) might have partially masked the negative sulfur odor. This might be a problem; however, further studies are needed to confirm that. The volatile compounds were positively correlated and to a great extent could explain the variation in the sensory odor attributes with significant differences, i.e., lemon, cinnamon, sulfur. High intensities of cinnamon and lemon odor were noticed from the chicken samples packed in active PLA_b_ composites during sensory analysis which might be a problem with respect to broader consumer acceptance. Future studies will focus on encapsulation of active components and their effect on the product quality.

## Figures and Tables

**Table 1:** List of ten packaging combinations (PC) consisting of five different packaging materials and two distinct gas compositions: MAP1 (60% CO_2_/40% N_2_) and MAP2 (75% O_2_/25% CO_2_).

Packaging Combinations (PC)	Material	Packing Method (MAP)-Gas Composition
1	PLA_b_	60% CO_2_/40% N_2_
2	PLA_b_	75% O_2_/25% CO_2_
3	PLA_b_ + 4%citral	60% CO_2_/40% N_2_
4	PLA_b_ + 4%citral	75% O_2_/25% CO_2_
5	PLA_b_ + 4%cinnamon oil	60% CO_2_/40% N_2_
6	PLA_b_ + 4%cinnamon oil	75% O_2_/25% CO_2_
7	PLA_b_ + 2%citral + 2%cinnamon oil	60% CO_2_/40% N_2_
8	PLA_b_ + 2%citral + 2%cinnamon oil	75% O_2_/25% CO_2_
9	Reference: APET/PE	60% CO_2_/40% N_2_
10	Reference: APET/PE	75% O_2_/25% CO_2_

**Table 2:** Definition of sensory attributes used in sensory profiling of raw chicken.

Odor Attribute	Description
Sour odor	Related to a fresh, balanced odor due to the presence of organic acids
Burnt odor	Associated with a burnt/burning odor
Metallic odor	Odor of metal (ferrous sulfate)
Sulfur odor	Odor of sulfur
Cinnamon odor	Associated to odor of spices (cinnamon, cloves, nutmeg)
Lemon odor	Associated to odor of lemon/ lemon aroma
Off odor	Related to odor which does not naturally exist in chicken
Cloying odor	Associated with an unfresh, sickening odor
Fermented odor	Associated to fermented acids, tainted
Rancid odor	The intensity of all rancid odors (grass, hay, candle, paint)
Prickly odor	Associated with a sharp, pungent odor

## Supplementary Materials

The following are available online at https://www.mdpi.com/article/10.3390/foods10051126/s1, Table S1. General linear model results, Table S2: Average values and *p*-values for each sensory attribute—appearance and texture; Table S3: Identified main volatile compounds in the empty packaging and their relative concentrations (area of the chromatographic peak) at the starting, 8th and 14th day of storage. Results are averaged from three parallel samples, Table S4: Detected major volatile compounds in the packaging with chicken samples and their relative concentrations (area of the chromatographic peak) at the 8th and 14th day of storage. Results are averaged from three parallel samples; Figure S1: Dry matter of chicken samples as a function of storage time for different packaging combinations, Figure S2: Bi-plot over samples and attributes—all attributes, O = Odour, T = Texture.
