# Supplementary material for: Biodegradable Active Packaging as an Alternative to Conventional Packaging: A Case Study with Chicken Fillets
Source: Foods. 2021 May 19;10(5):1126. doi: 10.3390/foods10051126 (PMC8161013; doi:10.3390/foods10051126)
Supplement: Supplementary file 1 [file foods-10-01126-s001.zip › foods-1157606-supplementary.pdf]

Supporting information

Table S1. General linear model results .

		Dip loss	Dry matter	TVC	LAB	STAA	VRBGA
**Material (tray)**	M	2.54	8.78	0.67	2.72	3.33	0.77
**Packaging Gas**	G	0.57	0.31	0.14	0.30	6.21	15.10
**storage time**	T	40.49	12.32	85.44	80.44	69.76	75.40
	MxG	7.09	2.20	4.25	6.39	0.73	1.94
	MxT	2.17	9.64	4.03	3.21	5.04	2.33
	GxT	2.18	1.50	0.09	0.06	0.28	1.99
	Error	44.96	65.26	5.37	6.88	14.65	2.46
	R-sq(adj)	42.07%	15.59%	93.05%	91.00%	80.83%	96.81%

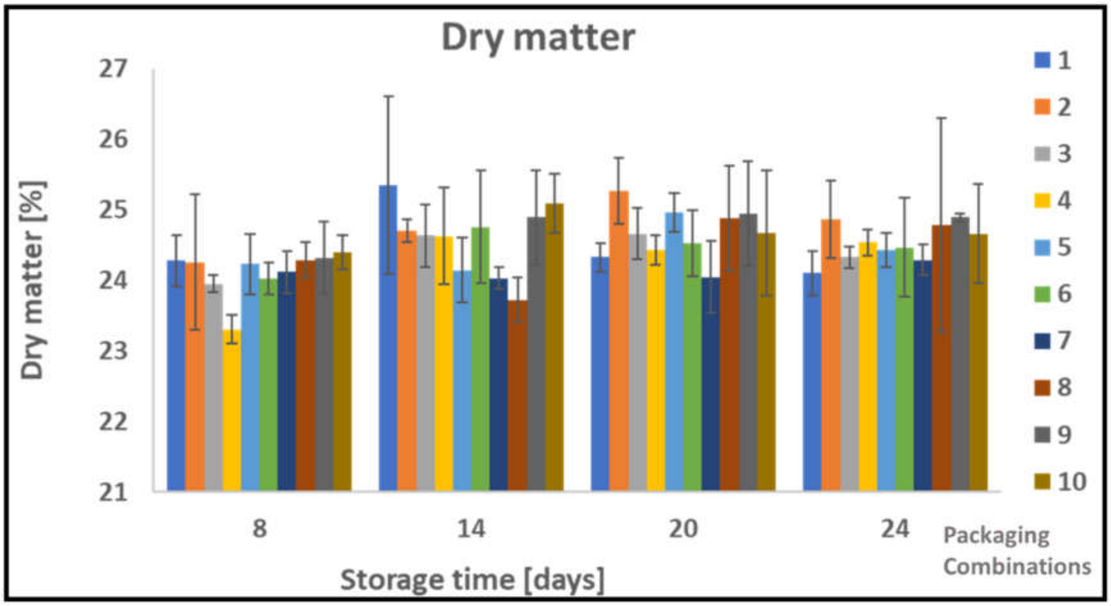

Figure S1: Dry matter of chicken samples as a function of storage time for different packaging combinations.

Table S2: Average values and p-values for each sensory attribute – appearance and texture

Samples from Packaging combinations	Colour hue	Colour intensity	Whiteness	Colour eveness	Glossiness	Dried performance (appenence)	Dried performance (texture)	Firmness
1.	6.18 AB	4.54 AB	5.36 A	5.56 A	6.21 AB	1.03 A	1.01 A	5.94 A
2.	5.90 ABC	4.26 AB	5.52 A	6.08 A	5.39 BC	1.13 A	1.09 A	6.20 A
3.	4.47 D	4.57 AB	5.19 A	4.84 A	6.25 A	1.16 A	1.22 A	5.26 A
4.	4.49 D	4.86 A	4.98 A	4.77 A	5.50 ABC	1.15 A	1.33 A	5.81 A
5.	6.02 ABC	3.99 B	5.69 A	5.88 A	6.32 A	1.11 A	1.06 A	5.76 A
6.	5.89 ABC	4.29 AB	5.53 A	5.42 A	5.25 C	1.08 A	1.01 A	5.66 A
7.	5.10 CD	4.37 AB	5.39 A	5.22 A	5.92 ABC	1.32 A	1.16 A	5.56 A
8.	5.27 BCD	4.48 AB	5.32 A	5.54 A	5.64 ABC	1.14 A	1.17 A	5.89 A
9.	5.66 ABC	4.44 AB	5.30 A	6.06 A	5.82 ABC	1.01 A	1.00 A	5.71 A
10.	6.37 A	4.71 AB	5.06 A	5.80 A	5.65 ABC	1.03 A	1.01 A	5.36 A
P- value	<0.001	0.026	0.054	0.099	<0.001	0.187	0.128	0.311

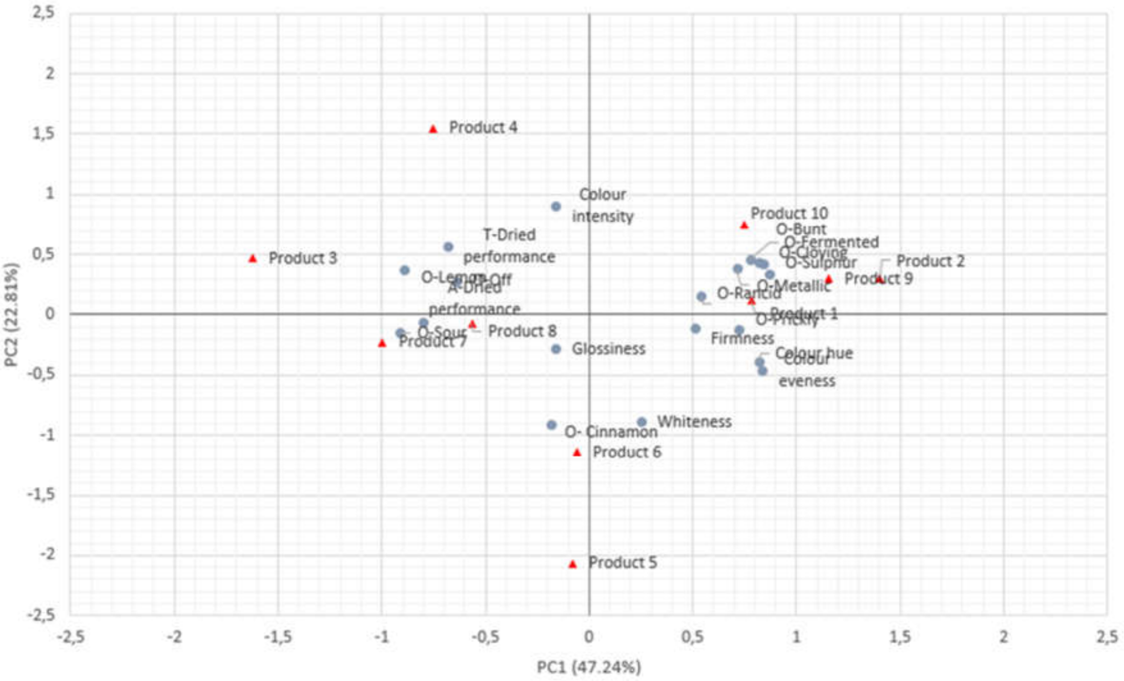

Figure S2: Bi-plot over samples and attributes - all attributes, O=Odour, T=Texture

Table S3: Identified main volatile compounds in the empty packaging and their relative concentrations (area of the chromatographic peak) at the starting, 8th and 14th day of storage. Results are averaged from three parallel samples.

	1-T-0 (×10^6)	1-T-8 (×10^6)	1-T-14 (×10^6)	3-T-0 (×10^6)	3-T-8 (×10^6)	3-T-14 (×10^6)	5-T-0 (×10^6)	5-T-8 (×10^6)
**1-Propene, 2-methyl-**							0.5549	
**n-Hexane**	0.7736	0.72063	1.16703				1.0345	1.14706
**2-Butene, 2-methyl-**				2.61876	3.0898	2.745845		
**1,3-Butadiene, 2-methyl-**				17.7247	4.9554	4.288678		
**Pentane, 2,2,4-trimethyl-**			1.56266		2.3624	2.146407		
**Hexane, 3-methyl-**				2.58466	2.8482		0.8341	1.09054
**Heptane**				1.87592	1.448	1.049834		
**Octane**				4.88684	2.9187	1.607139		
**Acetone**	2.1539	2.9695	2.69411	36.3499	49.542	39.4749	2.7226	2.17358
**3-(2-Cyclopenten-1-yl)propanal**				4.12178	3.2223	2.87022		
**Tetrahydrofuran**	347.56	322.918	296.031	297.848	160.93	96.28734	96.651	65.1044
**Cyclopentane, 2-ethyl-1,1-dimethyl**						20.52066		
**3-Nonene, (E)-**				26.1244	25.784	16.84861		
**2-Butenal**				195.993	84.162	45.72743		
**1-Tridecyne**				25.5884	20.534	14.61228		
**Furan, 3-methyl-**				7.89788	3.8733	2.270978		
**(3,3-Dimethyloxiranyl)methanol / Acetamide**				19.4942	12.418	6.989809		
**Ethanone, 1-(1-cyclohexen-1-yl)-**				5.94001	3.6967	2.643089		
**Isopropyl Alcohol**				8.03071	5.2774	3.575966		
**Cyclohexene, 1,6,6-trimethyl-**				6.06144	4.2338			
**Benzene**					4.6104	3.483673		
**Heptane, 2,2,4,6,6-pentamethyl-**	4.3768	4.44082	4.42121	4.21197	3.9565		5.7647	4.67197
**Methyl vinyl ketone**				14.1855	5.5863	4.39439		
**Cyclohexene,3-propyl-**				45.0432	20.7	17.2664		
**Ethanone, 1-(2-methyl-2-cyclopente n-1-yl)-**				17.5172	10.675	6.495139		
**1,5-Heptadiene, 2,6-dimethyl-**				21.1667	8.8833	6.503967		
**Tricyclo[2.2.1.0(2,6)]heptane, 1,7 ,7-trimethyl-**							11.479	10.4374
**(1S)-2,6,6-Trimethylbicyclo[3.1.1]hept-2-ene**				5.27466			1520.6	1321.69
**Bicyclo[3.1.0]hex-2-ene, 2-methyl-5-(1-methylethyl)-**							81.107	59.2019
**Toluene**	8.4713	5.11813	4.22962	18.8054	10.078	6.126193	16.134	9.23168
**Bicyclo[2.2.1]heptane, 7,7-dimethyl-2-methylene-**							21.319	18.4494

**3-Buten-2-ol, 2-methyl-**				500.321	271.69	147.832		
**Camphene**							526.35	465.262
**.beta.-Pinene**							330.44	286.573
**(+)-3-Carene**							88.695	67.6191
**.alpha.-Phellandrene**							757.35	538.148
**.beta.-Myrcene**							89.427	54.3758
**1,3-Cyclohexadiene, 1-methyl-4-(1-methylethyl)- / (+)-2-Carene**							123.82	83.0169
**Cyclopentanone**	63.486	45.3996	37.7423	52.786	30.736	21.10014	46.679	33.266
**2,3-Dehydro-1,8-cineole**				9.06971	6.5041	4.657915		
**D-Limonene**				25.5858	12.691	8.437234	247.37	168.612
**Eucalyptol**							110.66	97.8995
**.beta.-Phellandrene**							340.48	242.038
**2-Butenal, 3-methyl-**				14.2721	6.8797	4.476313		
**2,6-Dimethyl-1,3,5,7-octatetraene, E,E-**				6.35335	3.5019	3.399091		
**Cyclobutene, bis(1-methylethylidene)-**				9.88807				
**Butyl 2-methylbutanoate**				5.28733	3.4716		2.2701	
**(1R)-2,6,6-Trimethylbicyclo[3.1.1]hept-2-ene**							4.1279	2.38331
**.gamma.-Terpinene**							27.828	18.7284
**1,3,8-p-Menthatriene / Benzene, 1,4-diethyl- / Bicyclo[3.2.1]oct-2-ene, 3-methyl-4-methylene-**				16.7257	8.4728	7.173192		
**.beta.-Ocimene**							13.849	7.85726
**Styrene**	33.447	21.9914	17.5376	20.8834	10.538	7.376571	30.224	18.6788
**o-Cymene**				13.5942	6.7302	5.111848	606.76	427.025
**3-Carene**							7.5209	5.42402
**(+)-4-Carene**							50.64	34.6676
**5-Hepten-2-one, 6-methyl-**				10.2336	10.682	10.88084		
**1,3,8-p-Menthatriene**				2.29497		1.816453		
**Nonanal**							2.1878	2.34205
**Phenol, m-tert-butyl-**				2.86543	1.5902			
**Benzene, (2-methyl-1-propenyl)-o-Isopropenyltoluene**				11.3336		3.139442		
**Benzene, 1-methyl-4-(1-methylethen yl)-**				9.12544	4.545	4.29712	11.281	8.10744
**Acetic acid**	1.808	1.5571		18.0568	8.2228	4.513972	4.0845	2.85738
**Ethyl 2-(5-methyl-5-vinyltetrahydrofuran-2-yl)propan-2-yl carbonate**							4.0659	3.50296
**.alfa.-Copaene**				13.1847	7.6344	6.900076	94.028	94.5096
**3-Nonen-5-yne, 4-ethyl-, (Z)-**								
**Formic acid**				3.35547				

**(+)-2-Bornanone**							2.758	1.94493
**Benzaldehyde**	1.3892		1.2501	3.19762	1.5245	1.862566	29.016	20.3228
**1,6-Octadien-3-ol, 3,7-dimethyl-**				11.5246	1.97	2.706934	156.7	132.921
**Dimethyl Sulfoxide**								
**Pinocarvone**				2.20061				
**Bicyclo[7.2.0]undec-4-ene, 4,11,11-trimethyl-8-methylene-**							2.7452	2.25969
**Pulegone**				15.5021		7.376213		
**1H-Pyrrole, 2-ethyl-4-methyl-**					8.4928			
**Cyclohexane, 1-ethenyl-1-methyl-2, 4-bis(1-methylethenyl)-, [1S-(1.al pha.,2.beta.,4.beta.)]-**				8.25169	5.0409	4.689639		
**Caryophyllene**				28.7309	19.528	17.65576	188.55	195.162
**Terpinen-4-ol**							9.4855	8.59715
**Alloaromadendrene**				2.85523	1.7952	1.852218	4.6324	5.55091
**Acetophenone**				3.71816	2.091	1.356646	2.1881	1.74342
**Humulene**				4.78048	2.3705	2.184016	19.959	20.2854
**tr,Citral**				496.041	365.79	294.3516	1.5711	1.57797
**2-Carene**				7.88446	5.4738	4.249186	10.441	9.44769
**endo-Borneol**							2.6924	2.59979
**cis,Citral**				530.057	372.53	302.7714	2.866	2.41159
**2-Butenal, 3-methyl-**				9.60867	6.3719	6.604993		
**Furan, 2,3-dihydro-4-methyl-**					7.4404			
**2-Oxo-6-(piperidine-1-sulfonyl)-be nzooxazole-3-carboxylic acid 2-chloro-ethyl ester**					5.7928			
**Naphthalene, 1,2,3,5,6,8a-hexahydr o-4,7-dimethyl-1-(1-methylethyl)-,(1S-cis)-**							2.0176	1.94366
**Safrole**							15.964	11.5252
**Butylated Hydroxytoluene**								
**Hexane, 1-(1-methoxyethoxy)-**				1.64373				
**Epoxy-linalooloxide**								
**Phenol**				1.47307		1.237241	1.397	1.06314
**Cinnamaldehyde, (E)-**							5.5097	3.8318
**1,3-Cyclohexadiene-1-carboxaldehyde, 2,6,6-trimethyl-**				2.80461	1.4412	1.37144		
**Eugenol / 3-Allyl-6-methoxyphenol**				2.95068	2.2234	2.252182	135.14	118.08
**Benzoic acid**							1.5823	

	**5-T-14 $(\times 10^6)$**	**7-T-0 $(\times 10^6)$**	**7-T-8 $(\times 10^6)$**	**7-T-14 $(\times 10^6)$**	**9-T-0 $(\times 10^6)$**	**9-T-8 $(\times 10^6)$**	**9-T-14 $(\times 10^6)$**	
**1-Propene, 2-methyl-**	0.5994							
**n-Hexane**	0.7328			0.70061	1.0707			
**2-Butene, 2-methyl-**		0.9083		0.45004				
**1,3-Butadiene, 2-methyl-**		15.0587	8.10977	7.55893				
**Hexane, 3-methyl-**	1.0974	6.84972	8.30277	7.51163				
**Pentane, 3-ethyl-**		1.1643	1.36268	1.10837				
**Heptane**		2.08339	1.37878					
**Cyclopentane, 1,3-dimethyl-**		2.0843	2.10949	1.71695				
**Cyclopentane, 1,2-dimethyl-**		5.46859	5.66471	4.54719				
**Cyclohexane, methyl-**		10.5381	11.849	10.2426				
**Octane**		0.90044						
**Propanal, 2-methyl-**		2.34676	1.57397	1.37138				
**Acetone**	1.3557	36.6053	33.0998	30.7191				
**3-(2-Cyclopenten-1-yl)propanal**								
**Tetrahydrofuran**	43.31	196.479	152.069	107.248				
**3-Nonene, (E)-**								
**2-Butenal**		6.69452	4.55904	3.66366				
**1-Tridecyne**								
**Furan, 3-methyl-**		4.32927	1.03957					
**(3,3-Dimethyloxiranyl)methanol / Acetamide**		3.11794						
**Benzene**					3.1704			
**Heptane, 2,2,4,6,6-pentamethyl-**	4.2955	4.77518	4.02634	3.59421				
**Methyl vinyl ketone**		14.1414						
**1,3,5-Hexatriene, 3-methyl-, (Z)-**			8.04152	7.22648				
**Cyclohexene,3-propyl-**		9.51574	5.9601	5.11465				
**Ethanone, 1-(2-methyl-2-cyclopente n-1-yl)-**		3.56827	2.56821					
**1,5-Heptadiene, 2,6-dimethyl-**		5.23009						
**Tricyclo[2.2.1.0(2,6)]heptane, 1,7 ,7-trimethyl-**	8.1514	9.19344	7.19135	7.24911				
**(1S)-2,6,6-Trimethylbicyclo[3.1.1]hept-2-ene**	1037.8	1134.21	934.761	745.473				
**Bicyclo[3.1.0]hex-2-ene, 2-methyl-5-(1-methylethyl)-**	41.37	47.2464	32.5049	23.4992				
**Toluene**	5.5206	15.2062	8.41422	6.05595	4.3242	3.652667		
**Bicyclo[2.2.1]heptane, 7,7-dimethyl-2-methylene-**	13.435							
**3-Buten-2-ol, 2-methyl-**		280.489	182.961	122.091				

**Camphene**	361.8	369.877	305.686	248.114				
**.beta.-Pinene**	220.92	210.152	173.557	136.81				
**(+)-3-Carene**	47.341	50.7221	35.6877	26.6148				
**.alpha.-Phellandrene**	366.68	366.872	241.327	172.022				
**.beta.-Myrcene**	33.307	45.9326	25.8526	16.5277				
**1,3-Cyclohexadiene, 1-methyl-4-(1-methylethyl)- / (+)-2-Carene**	55.11	51.9364	31.7716	22.5915				
**Cyclopentanone**	22.214	49.7009	34.4203	25.0863				
**2,3-Dehydro-1,8-cineole**		4.09073						
**D-Limonene**	111.47	145.899	93.2055	67.0071				
**Eucalyptol**	75.46	63.7989	53.5102	43.7183				
**.beta.-Phellandrene**	168.83	163.938	110.919	81.7948				
**2,6-Dimethyl-1,3,5,7-octatetraene, E,E-**		19.6047	11.8214	9.77574				
**.gamma.-Terpinene**	12.537	13.2068	8.42322	6.21699				
**1,3,8-p-Menthatriene / Benzene, 1,4-diethyl- / Bicyclo[3.2.1]oct-2-ene, 3-methyl-4-methylene-**		25.1339	15.0327	12.6335				
**.beta.-Ocimene**	4.8719	6.79815	3.51441	2.42482				
**Styrene**	12.239	25.7554	15.2297	10.8953				
**o-Cymene**	293.53	354.88	227.841	169.333				
**3-Carene**	3.6404	3.68231						
**(+)-4-Carene**	23.157	25.1675	15.4655	11.1176				
**5-Hepten-2-one, 6-methyl-**		3.75639	3.0416	2.8956				
**1,3,8-p-Menthatriene**		3.42083	2.74011	2.11024				
**Nonanal**	2.3325							
**Phenol, m-tert-butyl-**		1.82435						
**Benzene, 1-methyl-4-(1-methylethen yl)-**	5.1026	16.5722	9.50141	8.33267				
**Acetic acid**	2.2005	6.62766	3.45122	2.53464	2.1833	1.588247	1.3903	
**Ethyl 2-(5-methyl-5-vinyltetrahydrofuran-2-yl)propan-2-yl carbonate**	2.6557	3.05287	2.49029	1.7538				
**.alfa.-Copaene**	75.546	55.2674	49.3667	43.6397				
**Formic acid**		2.46349						
**(+)-2-Bornanone**	1.8743							
**Benzaldehyde**	15.462	17.3362	11.8064	9.53553	1.3532	1.334138	1.2028	
**1,6-Octadien-3-ol, 3,7-dimethyl-**	97.842	85.6894	68.1327	52.5424				
**Pinocarvone**		1.1269						

**Bicyclo[7.2.0]undec-4-ene, 4,11,11-trimethyl-8-methylene-**				7.29648				
**Pulegone**		11.0556	8.97407					
**Cyclohexane, 1-ethenyl-1-methyl-2, 4-bis(1-methylethenyl)-, [1S-(1.al pha.,2.beta.,4.beta.)]-**		4.37808	3.92976	3.34354				
**Caryophyllene**	159.45	114.004	110.071	100.468				
**Terpinen-4-ol**	6.3006	5.26084	4.32369	3.53005				
**Alloaromadendrene**	4.8371	4.50647	4.06686	3.4355				
**Acetophenone**	1.8827	1.45103	1.26986	1.21628	1.1856			
**Humulene**	15.762	9.51203	8.92919	9.67264				
**tr. Citral**	0.9708	332.905	267.301	207.181				
**2-Carene**	6.6037	9.45166	7.81967	6.37981				
**endo-Borneol**	1.9193	2.53378	2.17321	2.46698				
**cis, Citral**	1.8513	368.949	288.097	223.196				
**2-Butenal, 3-methyl-**		5.20555						
**Naphthalene, 1,2,3,5,6,8a-hexahydr o-4,7-dimethyl-1-(1-methylethyl)-,(1S-cis)-**	1.8963							
**Safrole**	8.2081	8.23225	5.46296	4.32224				
**Butylated Hydroxytoluene**					1.3492	1.463919	1.28	
**Phenol**	1.2602	1.96358	1.58642	1.36696				
**Cinnamaldehyde, (E)-**	2.8429	2.98858	2.049	1.53936				
**1,3-Cyclohexadiene-1-carboxaldehyde, 2,6,6-trimethyl-**		2.19312	1.52085	1.2719				
**Eugenol / 3-Allyl-6-methoxyphenol**	90.858	68.5104	56.8758	45.503				
**Benzoic acid**		2.04274						

Table S4: Detected major volatile compounds in the packaging with chicken samples and their relative concentrations (area of the chromatographic peak) at the 8th and 14th day of storage. Results are averaged from three parallel samples.

	**1-T-8 $(\times 10^6)$**	**1-T-14 $(\times 10^6)$**	**2-T-8 $(\times 10^6)$**	**2-T-14 $(\times 10^6)$**	**3-T-8 $(\times 10^6)$**	**3-T-14 $(\times 10^6)$**	**4-T-8 $(\times 10^6)$**	**4-T-14 $(\times 10^6)$**	**5-T-8 $(\times 10^6)$**	**5-T-14 $(\times 10^6)$**
**1-Propene, 2-methyl-**					3.307	3.0671	3.128	3.1855	0.453	
**Pentane**	0.97916	1.3506		0.71028	1.151	1.4352		0.5776		0.543
**n-Hexane**		0.8485		0.80631			1.54	2.3882		0.643

**2-Butene, 2-methyl-**					2.159	2.2904	1.376	1.9758		
**1,3-Butadiene, 2-methyl-**					1.932	1.6139	1.219	1.2509		
**Pentane, 2,2,4-trimethyl-**								2.1276		
**Hexane, 3-methyl-**					2.204	2.519	1.439	1.8531	0.976	1.075
**Heptane**					1.058	1.1444	0.811			
**Carbon disulfide**	2.31494	1.6086	2.6609	1.53498	1.664	1.0302	2.477	1.8849	1.826	1.421
**Dimethyl sulfide**	4.8014	16.951	7.1598		4.49	3.9051	6.623		11.26	5.049
**Octane**					1.461	1.1079	1.23			
**Acetone**	8.02099	7.1833	7.6172	6.95285	16.6	19.138	14.84	17.792	6.651	6.167
**3-(2-Cyclopenten-1-yl)propanal**					1.868	1.7861				
**Tetrahydrofuran**	9.204	6.5329	4.0977	4.62634	6.258	6.008	5.045	4.9107	5.126	4.117
**Cyclopentane, 2-ethyl-1,1-dimethyl**					5.899	6.1305	5.038	4.498		
**3-Nonene, (E)-**							5.6			
**1-Tridecyne**					5.477	5.0542	4.383	3.6994		
**Furan, 3-methyl-**					2.903	2.5623	2.964	2.5807		
**Isopropyl Alcohol**						4.5507		4.5903		
**Ethanol**						1.6441		2.2963		
**Benzene**							3.333			
**Cyclohexene,3-propyl-**					10.31	9.4209	10.01	7.0748		
**Ethanone, 1-(2-methyl-2-cyclopente n-1-yl)-**					3.823	4.279	3.55	3.2539		
**1,5-Heptadiene, 2,6-dimethyl-**					4.129	3.7736	4.315	4.1098		
**Tricyclo[2.2.1.0(2,6)]heptane, 1,7 ,7-trimethyl-**									4.068	
**(1S)-2,6,6-Trimethylbicyclo[3.1.1]hept-2-ene**									564.4	500.5
**Bicyclo[3.1.0]hex-2-ene, 2-methyl-5-(1-methylethyl)-**									25.3	19.08
**Toluene**									5.756	4.248
**Bicyclo[2.2.1]heptane, 7,7-dimethyl-2-methylene-**									5.266	4.943
**3-Buten-2-ol, 2-methyl-**						4.3483				
**Camphene**									145.7	134.2
**.beta.-Pinene**									91.9	79.37
**(+)-3-Carene**									25.72	20.11
**.alpha.-Phellandrene**									256.9	190.1
**.beta.-Myrcene**									26.28	18.7
**1,3-Cyclohexadiene, 1-methyl-4-(1-methylethyl)-/ (+)-2-Carene**									35.16	26.3
**D-Limonene**			5.5083		6.838	5.8856	7.539	4.9148	81.27	61.47
**Eucalyptol**									4.969	4.224
**.beta.-Phellandrene**									104.9	79.68
**Cyclobutene, bis(1-methylethylidene)-**						3.0145				

**.gamma.-Terpinene**									8.071	5.609
**1,3,8-p-Menthatriene / Benzene, 1,4-diethyl- / Bicyclo[3.2.1]oct-2-ene, 3-methyl-4-methylene-**					4.356	4.1848	3.574	3.4361		
**.beta.-Ocimene**									4.026	2.606
**Styrene**	8.47534	5.6956	6.3347	5.40617	5.192	4.4747	5.453	4.3488	10.3	8.033
**o-Cymene**					3.228	2.7271	2.995	2.5092	198.2	157.7
**3-Carene**									2.844	
**Cyclohexene, 1-methyl-4-(1-methyle thylidene)-**										8.571
**(+)-4-Carene**									13.19	9.085
**5-Hepten-2-one, 6-methyl-**					2.489	3.6023	3.32	5.2373		
**Nonanal**					1.837					
**Benzene, (2-methyl-1-propenyl)-o-Isopropenyltoluene**					2.709	2.248				
**Benzene, 1-methyl-4-(1-methylethen yl)-**							2.083	2.4523	2.07	1.902
**Acetic acid**		1.2544	1.5114	2.02384	2.599	1.5752	1.598	1.9778	1.247	1.446
**.alfa.-Copaene**					2.213	2.1868	2.231	2.2399	30.22	26.57
**3-Nonen-5-yne, 4-ethyl-, (Z)-**					2.744	4.5119		2.7965		
**Benzaldehyde**	1.23113	1.5726	1.3221	1.64815	1.83	2.0619	1.713	2.0998	2.608	1.786
**1,6-Octadien-3-ol, 3,7-dimethyl-**									26.81	22.99
**Dimethyl Sulfoxide**	1.93159	6.1066	2.7283		2.44	2.6109	4.141		2.402	1.382
**Pulegone**										
**1H-Pyrrole, 2-ethyl-4-methyl-**					2.347	5.4131	2.22	3.3271		
**Cyclohexane, 1-ethenyl-1-methyl-2, 4-bis(1-methylethenyl)-, [1S-(1.al pha.,2.beta.,4.beta.)]-**					1.754	2.0665	2.055	2.1275		
**Caryophyllene**					5.947	6.9283	6.948	7.1725	71.5	67.62
**Terpinen-4-ol**									0.775	0.708
**Alloaromadendrene**									2.476	2.183
**Acetophenone**				1.20848		1.1192	1.036	1.2982	1.265	1.245
**Humulene**									6.976	6.857
**tr, Citral**					94.71	99.753	104.8	93.085		
**2-Carene**						2.3573			2.224	2.095
**cis, Citral**					139.5	145.35	150.4	141.01		
**2-Butenal, 3-methyl-**										
**Furan, 2,3-dihydro-4-methyl-**					1.855	2.4486	2.519	2.2294		
**2-Oxo-6-(piperidine-1-sulfonyl)-be nzooxazole-3-carboxylic acid 2-chloro-ethyl ester**						1.7284	1.737	1.7333		

**Safrole**									6.4	6.362
**Hexane, 1-(1-methoxyethoxy)-**					1.849	2.544				
**Epoxy-linalooloxide**						2.0489		1.571		
**Phenol**						1.0863		1.2198		
**Cinnamaldehyde, (E)-**									1.71	1.725
**1,3-Cyclohexadiene-1-carboxaldehyde, 2,6,6-trimethyl-**						1.2203				
**Eugenol / 3-Allyl-6-methoxyphenol**					1.183	1.5706			51.84	59.15
**Benzoic acid**								1.6459		

	**6-T-8 $(\times10^{6})$**	**6-T-14 $(\times10^{6})$**	**7-T-8 $(\times10^{6})$**	**7-T-14 $(\times10^{6})$**	**8-T-8 $(\times10^{6})$**	**8-T-14 $(\times10^{6})$**	**9-T-8 $(\times10^{6})$**	**9-T-14 $(\times10^{6})$**	**10-T-8 $(\times10^{6})$**	**10-T-14 $(\times10^{6})$**
**1-Propene, 2-methyl-**	0.44116	0.4952	0.9754	0.85998		0.8552			0.354	0.393
**Pentane**			0.5594	0.66081				0.5673		
**n-Hexane**		0.7915				1.6393				
**2-Butene, 2-methyl-**			1.4494	1.35867	0.888					
**1,3-Butadiene, 2-methyl-**			1.6255	1.51267	1.326	1.3529				
**Pentane, 2,2,4-trimethyl-**										
**Hexane, 3-methyl-**	1.03387	1.2668	2.2453	2.30716	1.795	2.2344				
**Heptane**										
**Carbon disulfide**	1.37776	0.9241	1.532	1.01913	1.453	1.0482	3.807	3.5192	3.122	3.328
**Dimethyl sulfide**	1.43428		6.0022	5.78821			8.674	11.05		
**Octane**										
**Acetone**	6.44546	5.961	8.0983	8.82527	7.473	9.2264	6.137	5.8435	7.019	6.521
**3-(2-Cyclopenten-1-yl)propanal**										
**Tetrahydrofuran**	4.94868	4.4141	5.0356	5.81674	4.826	5.4038				
**Isopropyl Alcohol**										2.207
**Ethanol**										1.482
**Cyclohexene,3-propyl-**			2.8862	2.53854	2.818					
**Tricyclo[2.2.1.0(2,6)]heptane, 1,7 ,7-trimethyl-**	3.85789	3.9482								
**(1S)-2,6,6-Trimethylbicyclo[3.1.1]hept-2-ene**	580.029	462.98	335.36	372.863	326.9	326.76				
**Bicyclo[3.1.0]hex-2-ene, 2-methyl-5-(1-methylethyl)-**	25.1591	18.317	12.285	12.1628	12.25	9.933				
**Toluene**	5.13705	4.5348	4.1653	4.03992	4.055	3.7362				
**Bicyclo[2.2.1]heptane, 7,7-dimethyl-2-methylene-**	5.59797	5.7175								
**Camphene**	150.755	123.84	83.03	95.3635	81.66	83.54				
**.beta.-Pinene**	93.6364	73.669	47.37	53.2973	47.55	47.408				

**(+)-3-Carene**	25.579	17.98	12.125	12.3757	12.59	11.012				
**.alpha.-Phellandrene**	251.574	173.42	101.35	95.3462	107	85.276				
**.beta.-Myrcene**	26.3131	16.808	11.633	9.96825	12.46	9.053				
**1,3-Cyclohexadiene, 1-methyl-4-(1-methylethyl)- / (+)-2-Carene**	34.6182	23.82	12.742	11.8567	13.41	10.46				
**D-Limonene**	79.3087	54.469	42.211	38.778	45.1	35.297		3.9819		
**Eucalyptol**	4.75418	3.9457								
**.beta.-Phellandrene**	102.665	71.932	44.012	41.5536	45.78	37.388				
**2,6-Dimethyl-1,3,5,7-octatetraene, E,E-**			4.6744	3.81501	4.727	3.1053				
**.gamma.-Terpinene**	7.93208	5.1019	3.2186	3.19232	3.472	2.8408				
**1,3,8-p-Menthatriene / Benzene, 1,4-diethyl- / Bicyclo[3.2.1]oct-2-ene, 3-methyl-4-methylene-**			6.0275	5.05602	6.104	4.2518				
**.beta.-Ocimene**	3.99987	2.4964								
**Styrene**	9.46618	7.1075	7.7903	7.0493	8.192	6.7049				
**o-Cymene**	193.932	141.54	103.98	95.2907	105.3	88.334				
**3-Carene**										
**Cyclohexene, 1-methyl-4-(1-methyle thylidene)-**	12.9154	7.6658	5.3909	4.40811	5.408	3.9107				
**5-Hepten-2-one, 6-methyl-**					1.514	1.6769				
**Nonanal**							1.497			1.386
**Benzene, 1-methyl-4-(1-methylethen yl)-**	1.9194	1.4016	3.2862	2.73702	3.356	2.5059				
**Acetic acid**	1.22211	1.7827	1.3886	1.87732		1.7887	1.291	1.3878		1.888
**.alfa.-Copaene**	28.3629	21.404	13.061	14.0254	12.93	13.305				
**Benzaldehyde**	2.13304	1.6871	2.5323	2.50366	1.848	2.4324	1.287	1.4588	1.372	1.494
**1,6-Octadien-3-ol, 3,7-dimethyl-**	25.7154	20.667	13.547	11.0673	11.75	8.6144				
**Dimethyl Sulfoxide**	1.63754		3.2339	2.72522			2.068	2.4157		
**Pulegone**			1.8563		2.132					
**Cyclohexane, 1-ethenyl-1-methyl-2, 4-bis(1-methylethenyl)-, [1S-(1.al pha.,2.beta.,4.beta.)]-**			1.4097	1.31355	1.226	1.5527				
**Caryophyllene**	71.1304	57.709	33.067	37.0886	33.18	36.524				
**Terpinen-4-ol**	0.90424	0.6654								
**Alloaromadendrene**	2.16524	2.117				1.6157				
**Acetophenone**	1.30031		1.1195	1.18377		1.2781				1.183
**Humulene**	7.59758	6.2935	2.8021	3.05545	3.061	2.9376				
**tr, Citral**			83.723	59.0131	65.29	41.352				
**2-Carene**	2.14772	1.9502	2.42	2.02493	2.109	1.7457				

**cis,Citral**			121.57	88.1894	100.3	67.624	1.221	2.058		
**2-Butenal, 3-methyl-**					0.918					
**Furan, 2,3-dihydro-4-methyl-**			1.4388	0.88086	1.427	0.8224				
**Safrole**	7.85689	6.7379	3.3439	2.60822	3.347	2.5941				
**Phenol**			1.1047			1.215				
**Cinnamaldehyde, (E)-**	1.97868	1.6421	1.235							
**Eugenol / 3-Allyl-6-methoxyphenol**	66.998	68.504	26.456	21.1191	31.58	21.524				
**Benzoic acid**						0.6873				
